# Seasonal Variation of Chemical Composition, Fatty Acid Profile, and Sensory Properties of a Mountain Pecorino Cheese

**DOI:** 10.3390/foods9081091

**Published:** 2020-08-10

**Authors:** Francesco Serrapica, Felicia Masucci, Antonio Di Francia, Fabio Napolitano, Ada Braghieri, Giulia Esposito, Raffaele Romano

**Affiliations:** 1 Dipartimento di Agraria, Università di Napoli Federico II, Via Università 100, 80055 Portici, Italy; francesco.serrapica@unina.it (F.S.); antonio.difrancia@unina.it (A.D.F.); raffaele.romano@unina.it (R.R.); 2 Scuola di Scienze Agrarie, Forestali, Alimentari ed Ambientali, Università degli Studi della Basilicata, Via dell’Ateneo Lucano 10, 85100 Potenza, Italy; fabio.napolitano@unibas.it (F.N.); ada.braghieri@unibas.it (A.B.); 3 Department of Animal Sciences, Faculty of AgriSciences, Stellenbosch University, Matieland 7602, South Africa; giulia@sun.ac.za; 4 Research and Development RUM & N Sas, via Sant’Ambrogio 4/A, 42123 Reggio Emilia, Italy

**Keywords:** pecorino cheese, pasture, management system, fatty acids profile, sensory properties, consumer liking

## Abstract

This study aims to assess the compositional traits and sensory characteristics of a traditional pecorino cheese associated with management and feeding system seasonality. The study was carried out on two mountain dairy farms using an outdoor, pasture-based system from April to October (OutS), and an indoor system (InS) during the rest of the year. Outdoor-produced milk had higher fat content and a tendency for protein and somatic cell count to be higher. The OutS cheeses showed higher dry matter and fat content, higher percentages of unsaturated fatty acids, C18:3, *cis*-9, *trans*-11 conjugated linoleic acid, and *trans*-11 C18:1, and lower percentages of C14:0 and C16:0. These modifications in fatty acid composition determined the reduction of the atherogenic index. The OutS cheeses also displayed higher intensity of almost all sensory attributes, including odor, flavor, taste, and texture descriptors. The outdoor system partly reduced the liking of consumers for pecorino. However, changes in the productive process leading to an increment in the water content and softness of the cheeses (i.e., controlled humidity and temperature during ripening) may increase the overall liking of pasture-based products, thus promoting the consumption of healthier foods.

## 1. Introduction

Locally produced traditional foods are positively perceived by consumers and, although a role is played by consumer feelings such as nostalgia, ethnocentrism, and need of authenticity, sensory properties remain the main determinant of purchase intent and food liking [1]. The sensory properties of cheese can be affected by several environmental and technological factors [2,3]. In particular, feeding plays a central role in affecting milk characteristics, which in turn shape cheese quality traits [4]. Forages, which represent the main ingredient of ruminants’ diets, are able to convey specific nutritional and organoleptic features to milk. Thus, they can significantly contribute to define cheese “terroir” and healthiness [5]. Several studies have focused on the effect of botanical diversity and preservation methods of forage (fresh, hay, or ensiled) on the quality of various cheeses [6,7,8,9]. In particular, it has been well established that pasture and fresh herbage can positively change the fatty acid (FA) composition of milk fat in terms of polyunsaturated fatty acids (PUFA). The latter are transferred into the corresponding dairy products affecting cheese flavor and texture [10].

In many non-irrigable inland areas of the European Mediterranean regions, pasture-based sheep farming sustains the socio-economic vitality of local rural communities, also preventing environmental damage and changes in the traditional agricultural landscape [11]. In the regions of southern Italy, the extensive and semi-extensive sheep farms are mainly devoted to produce light lambs (22–24 kg live weight) as well as milk processed in several cheese varieties linked to the local traditions, generically named pecorino. In these areas, the pastures are characterized by a marked seasonal fluctuation of botanical composition and productivity [12]. The vegetative cycle begins in autumn, as the first rains appear, passes through a winter dormancy period imposed by the low temperatures, and then reaches maximum vegetative vigor in the late spring and early summer due to the favorable combinations of temperature and rainfall [12]. Consequently, the farming system seasonally changes from extensive into semi-extensive or indoors and, accordingly, from pasture grazing to diets based on preserved forages. Changes in ewe milk yield and composition associated with seasonal variations of feeding regimes have been addressed by a large body of literature, and the effects on physiochemical characteristics and fatty acid profiles of milk have been reviewed [13,14]. Nevertheless, studies addressing seasonal variation on pecorino cheese characteristics are scanty [15,16,17], and none of them have examined the impact of feeding regime on cheese sensory and chemical properties.

Thus, this study aimed to assess in Bagnolese pecorino (a cheese recognized as a Traditional Agri-Food Product by the Italian Ministry of Agriculture) the compositional traits and sensory characteristics associated with management and feeding system seasonality. In addition, the consumer liking for cheeses made by different dairies and in different systems was evaluated.

## 2. Materials and Methods

### 2.1. Study Site

The study was conducted at two family-owned, small-scale dairy sheep farms (A and B) located in an internal upland area (41°00′ N 15°26′ E and 41°01′ N 15°32′ E; about 900 m above sea level) of Campania, a Region of southern Italy. The farms were selected based on two main criteria: (1) use of pasture as the main feeding source during the grazing season; and (2) Bagnolese pecorino cheese entirely produced from farm milk. Farming was based on a mixed cereals–semi extensive sheep production system. The available dry arable land was used for cropping winter cereals and mixed meadows for hay production. The grazing pastures were private, and the flocks were moved to different grazing areas when forage was considered scarce. Both farms raised the native sheep breed “Bagnolese”, averaging three lambings every two years. Lambs were milk-fed until slaughter, at about 30–40 days of age, and ewes were milked thereafter, once a day, at evening. From April to October the flocks were kept on pasture integrated in the case of lactating animals with hay and concentrate mixture. Ewes were reared indoors during the rest of the year, when they were fed hay and cereal grains. Semi-arid grassland providing annual herbaceous plants, shrub pastures and, during drought, summer stubbles were the resources available for grazing. The main farm characteristics are reported in Table 1.

### 2.2. Experimental Design and Sampling Procedure

According to the pasture availability, the management system, and the season, the study was divided into two periods that are designated hereafter as Outdoor (OutS) and Indoor systems (InS). The InS period was from January to February, and the OutS period from April to May. During each period, each farm was visited at 2-week intervals (3 visits/farm/period). In the OutS period, the grass was sampled from movable grazing exclusion cages (1.5 m × 1.5 m) randomly located in the pasture before the grazing season, and in both OutS and InS periods the feeds fed in the barns were collected, and the bulk milk (200 mL) was gathered before cheese making.

The pecorino cheese manufacturing process at the artisanal dairy plants adjoining the farms was followed at each visit. The two dairies used similar cheese-making processes to produce the local semi-cooked cheese “Bagnolese pecorino”. Briefly, filtered raw ewe milk was gently heated in a copper vat to 36–38 °C, and kid paste rennet was added. At curd formation, the coagulum was cut by a wooden stick to rice size, cooked at 42 C° for about 5 min, and transferred into perforated plastic molds (about 2 L in volume) and pressed by hand to allow the whey drainage. Molded curd was left to acidify overnight and then dry salted by hand. The pecorino cheeses produced from the sampled milk were marked so as to be identified later. These cheeses were air-ripened in cool farm cellars for 60 d with frequent rotations and cleaning of the surface and then sent to the laboratory for analyses.

### 2.3. Analysis

#### 2.3.1. Chemical and Instrumental Analysis of Milk and Cheese

The samples of hay (*n* = 6, 2 farms × 3 sampling times) and pasture (*n* = 18, 2 farms × 3 sampling time × 3 cages) were dried in a forced-air oven (at 65 °C until constant weight), ground to pass a 1-mm screen, and then separately analyzed according the Association of Official Analytical Chemists (AOAC) for dry matter (DM), ash, ether extract, and crude protein [18]. The method of Van Soest et al. [19] was used to determine the neutral detergent fiber (NDF) and acid detergent fiber (ADF) contents. Energy content, expressed in MJ of net energy of lactation (NEL), was estimated according to the Sauvant and Nozière equation [20]. The milk fat and protein contents and somatic cell count (SCC) were determined by mid-infrared spectrophotometry (MilkoScan FT 6000 and Fossomatic 90, Foss Electric, Hillerød, Denmark). The chemical and fatty acid (FA) composition of pecorino was determined by sampling (about 300 g) each cheese (*n* = 6, 2 farms × 2 periods × 3 samples) at 1 cm from the rind. Quantification of moisture was performed by oven drying, and the fat and protein contents by the Gerber and Kjeldahl methods, respectively [18]. Fatty acid composition was determined after lipid extraction according the Schmidt–Bondzynski–Ratzlaff method, as reported by Romano et al. [21]. A Supelco 37 Component FAME mix (Supelco, Bellefonte, PA, USA) and a CLA (Conjugated Linoleic Acid) isomer mixture (Nu-Chek Prep. Inc., Elysian, MN, USA) were used as standards. Fatty acid values <0.1 were not quantified and atherogenic index was calculated [22]. Cheese color (CIELAB system) was measured on 3 non overlapping areas of a slice of cheese (Minolta colorimeter CR-300, Minolta Camera Co. Ltd., Osaka, Japan), as described elsewhere [23].

#### 2.3.2. Sensory Analysis and Consumer Liking of Pecorino Cheese

A quantitative–descriptive analysis (QDA) method [24] was used to assess pecorino cheese sensory properties. Sixteen subjects chosen among regular eaters of pecorino cheese (they had to consume pecorino cheese at least twice a month) were recruited by telephone. Among them, twelve panelists (average age 25 years) were selected following the recommendations issued by International Organization for Standardization (ISO) [25]. In particular, the sensitivity of the subjects to the four basic tastes was used [26]. During four preliminary sessions, the assessors developed a consensus list of attributes based on the available literature [27,28], resulting in a single score card with 2 odor, 2 flavor, 5 taste, and 5 texture descriptors (Table 2).

Subsequently, panelists were trained in the assessment of the intensity of sensory stimuli as suggested by Braghieri et al. [29]. Namely, the panel leader proposed a number of different products to be used as standard references for each attribute, and panelists identified which of them were the most appropriate. The completion of the entire reference frame, as reported in Table 2, required two 2 h sessions. In a further 2 h session, assessors were instructed to use the rating scale ranging from 0 (lack of sensation) to 100 (high intensity). For training purposes, at least two points of the scale were anchored using the previously identified references. In particular, panelists were informed about the intensity level of the samples to which they were exposed for three times. Then, they had to identify the intensity level of each attribute without any information. For this training eight 2 h sessions were required.

As to the actual QDA, in each session test, carried out at about 10.00 h, three rind-free cheese cubes (1 cm^3^) were served in random order and panelists gave a score between 0 and 100 for the intensity of each attribute. The panelists assessed five replications of each sample in sensory booths. In all, 5 sessions were conducted, and in each session two cheese cubes were offered in monadic randomized order and evaluated by each panelist when the temperature of each sample was 15 °C.

Pecorino cheeses were also evaluated for consumer liking [30] by 100 consumers balanced for sex with an average age of 40 years. Each participant evaluated four rind-free 1 cm^3^ cheese cubes (2 seasons by 2 replications). For each sample, consumers expressed an overall liking and a liking according to the following sensory inputs: appearance, texture, and taste/flavor. Consumers rated their liking on a hedonic scale ranging from “extremely unpleasant” (1) to “extremely pleasant” (9) with a central point (5) matching “neither unpleasant nor pleasant” [30].

### 2.4. Statistical Analysis

The SAS statistical software (SAS Institute Inc., Cary, NC, USA) was used to perform data analysis. Data on milk and cheese composition were analyzed by two-way analysis of variance (GLM procedure) to determine the fixed effect of management system (OutS and InS), farm (A and B), and their interaction (management system by farm). The sensory attributes of pecorino cheese were analyzed by ANOVA with assessor (12), replication (5), product (4 = 2 management system × 2 farms), and their interactions as factors. In addition, an analysis of variance with management system (2), farm (2), and their interaction as factors was conducted. Similarly, consumer liking was subjected to analysis of variance using management system (2), farm (2), and their interaction as factors.

## 3. Results and Discussion

### 3.1. Pasture Composition

The grazing pastures of both farms were mostly composed of Poaceae and Fabaceae with reduced incidence of other grazing species (Table 3), since they were sown at 3–4 year intervals with the grass–legume mixtures as usually managed in the investigated area [31,32].

### 3.2. Milk Traits and Cheese Chemical Composition and Color

The chemical composition of milk and cheese produced in the two farms as influenced by management system are given in Table 4. In agreement with previous studies [16,33], the OutS milk showed higher (*p* < 0.05) fat content, and a tendency (*p* < 0.1) for protein and SCC to be higher. Due to the market demand for young lambs at Christmas [34,35], in both flocks most of the ewes lambed in autumn, and only primiparous and a small percentage of pluriparous animals (about 20%) lambed at the end of winter. Thus, the observed increments of milk components may be due to the concentration effect related to the lower yield at the end of lactation [16,36]. Moreover, also the progressive deterioration of the ewe udder health in late lactation can increase SCC in ewe milk [37,38]. The proximate chemical composition of pecorino cheese was within the standards required for similar ewe cheeses endowed by protected denomination origin, such as Canestrato Pugliese cheese and the related production rules (e.g., fat content not less than 38% in DM). The cheese OutS showed a higher DM content (*p* < 0.01), indicating a faster loss of moisture during the aging period in summer, and a higher fat percentage (*p* < 0.001), likely related to the tendency observed in OutS milk fat to be higher. Except for the ash content, significant effects of the farm were observed for all the chemical characteristics of pecorino cheese. These differences are expected for traditional cheese produced at small scale dairies [16]. Unsurprisingly, neither farm nor management system affected cheese color. Previous studies reported changes in cheese instrumental color as a consequence of using fresh forage and the closely related β-carotene content [31,39,40,41]. However, β-carotene is not detectable in ewes’ milk due to its enzyme-catalyzed cleavage to retinal in the sheep liver [42]. As a consequence, pecorino cheese color may be unaffected by changes in feeding and β-carotene intake.

Fatty acid composition of pecorino cheese is given in Table 5. As to the neo-formed (C ≤ 15) and mixed (C16:0 and C16:1) FA [43], the OutS cheeses showed lower values of C12:0 (*p* < 0.05) and C16:0, C14:0 (*p* < 0.001), whereas the C4:0 content was tendentially higher (*p* < 0.1). A reduced synthesis of short and medium-chain FA in the mammary gland may be observed in lactating ruminants on pasture-based diets, due to either high levels of dietary PUFA from fresh forage that compete with de novo FA for the esterification in the mammary gland, or the negative energy balance that may often occur when milk yield is high [10]. Nevertheless, a lack of a marked reduction of de novo FA in animals fed fresh forage has been also reported [31]. In the current work, the low nutrient requirements of the ewes in late stage of lactation might explain the lack of the effect of management system on a number of de novo FA (i.e., C6:0, C8:0, C10:0; C14:1, C15:0). In agreement with numerous authors [43,44,45], OutS cheeses also showed higher levels of the dietary origin of FAs C18:1 n9*cis*, C18:2 n6 *cis*, C18:1 *trans* 11, (*p* < 0.001), C18:3 n3 (*p* < 0.01), *cis*-9,*trans* 11 conjugated linoleic acid (CLA), and C18:1 *trans*-9 (*p* < 0.05). As an overall result, it was possible to recognize the effect of grazing on cheese FA composition in terms of increased content of PUFA (*p* < 0.001), *cis*-9,*trans*-11 CLA, and *trans*-11 C18:1 along with a reduction of SFA and atherogenic index (*p* < 0.001) under real flock management conditions.

A large body of literature (Mele [46], Nudda et al. [13], and Cabiddu et al. [14]) reports a “seasonal” effect on the FA composition of the milk fat, with an increase of the concentration of polyunsaturated PUFA and CLA in spring, due to the corresponding increment in the content in polyunsaturated FA in fresh forage, including C18:3. In addition, the highly degradable nitrogen and fiber of fresh forage along with a higher rumen pH related to grazing activity may promote the rumen bacteria producing CLA *cis*-9,*trans*-11 while inhibiting the biohydrogenation of C18:1 *trans*-11, which is converted to CLA *cis*-9,*trans*-11 via Δ9-desaturase in the mammary gland [47]. Although several studies investigated FA composition of ewe milk fat as influenced by the season of production, the literature on pecorino cheese is limited [15,16,17,48,49,50,51]. The use of vegetable seeds and oils [52,53,54,55,56,57] led to changes in fatty acid composition of ewe milk and cheese higher than those we observed. According to Dewhurst et al. [47] pasture is less efficient than oil or concentrates for modifying milk FA, but, on the other hand, it does not impact on feeding costs, and contributes to the basis for the “terroir” notion [6]. In addition, consumers are interested in traditional cheese with healthier FA profile, but without a substantial increase of the purchase price [58]. The farm of production slightly influenced the acidic composition of the cheese. This result can be explained based on the similar winter-feeding regimen (i.e., hays and grain meals) and similar pasture composition (i.e., semi-natural sown pasture) of the two farms, according with the results of other on-farm studies reporting that differences in milk FA composition are mainly related to the diet and pasture botanical composition [31,59,60,61].

### 3.3. Cheese Sensory Properties and Consumer Liking

An important outcome to emerge from this study is represented by the lack of any significant interactions of product with both replication and assessor. This finding suggests that the panel was subjected to an effective training as the products were consistently evaluated across replications and assessors. Based on these preliminary results, we conducted a second analysis of variance with farm, management system, and their interaction as factors. The second analysis showed that several sensory properties of pecorino cheese were affected by the management system (Table 6) as only the taste attributes “acid” and “bitter” and the texture attribute “solubility” were unaffected. Overall, the OutS cheeses showed a higher intensity of almost all sensory attributes. In particular, the trained panel perceived a higher intensity of the odor attributes “barn” (*p* < 0.001) and “hay” (*p* < 0.05), as well as a higher intensity of the flavor attributes “barn” (*p* < 0.01) and “pecorino” *(p* < 0.001) in OutS cheeses than InS. OutS cheeses were also perceived as more “salty” (*p* < 0.001) and “spicy” (*p* < 0.05) and less “sweet” (*p* < 0.001) than InS in terms of taste attributes. In addition, OutS cheeses showed higher intensities of the texture attributes “hardness”, “friability”, and “graininess” (*p* < 0.001), whereas they had a lower intensity of adhesivity (*p* < 0.05). The literature regarding cheese produced from animals on pasture does not provide unequivocal indications as far as sensory properties are concerned, since the cheese making procedure, including the use of starter cultures and the ripening conditions, can markedly influence the sensory properties of cheeses [62,63], thus possibly confounding the effects of diet on milk components. However, when the cheese-making process is conducted under controlled conditions, some cheese sensory properties may be traced back to the diet [6]. Pasture is rich in odor-active compounds that can be transferred to cheese [39]. In addition, the higher amount of MUFA and PUFA produced in pasture-based systems can influence the development of compounds which are active in terms of flavor and odor, and that is particularly so in aged cheese [64]. Nevertheless, conflicting results are reported on the effect of grazing on odor/flavor attributes. In agreement with our results, Cantal cheese produced from grazing pasture showed higher intensities of overall odor and aroma compared with the indoor system [65]. Conversely, a lower intensity of odor as a consequence of the ingestion of fresh forage was observed in ripened and fresh pasta filata cheeses [7,31]. These contrasting results may likely to be due to differences in the cheese making process. Both Bagnolese pecorino and Cantal are semi-cooked cheeses produced from raw milk so that neither milk nor curd are subjected to temperatures higher than 50 °C. By contrast, during pasta filata cheese making, the curd is stretched in water or whey heated at 80–90 °C. This thermal treatment may at least partly reduce the activity of the odor compounds in the milk obtained from grazing animals thus flattening the odor profile of the corresponding cheese. Accordingly, it has been recently observed that the amount of volatile organic compounds of milk may be markedly reduced in mozzarella, due to the high temperatures of curd during stretching [66].

In agreement with McSweeney [67], in summer a higher ripening temperature may have induced higher proteolysis levels with the production of amino acids and short peptides responsible for a higher intensity of the attribute “spicy” in OutS cheeses. Similarly, in the same products (i.e., OutS cheeses) a higher water loss related to a higher ripening temperature may have also caused an increase in the intensity of the taste attribute “salty” and a corresponding reduction of the intensity of the attribute “sweetness”. As to texture attributes, higher intensity values of “hardness” were unexpectedly perceived in OutS cheese as compared with InS cheese. As also reported in previous studies, a profile richer in unsaturated FAs, which are characterized by a lower melting point, may lead to softer and spreadable cheeses [6,7]. However, our results are not necessarily in contrast with those reported in previous studies. They may be rather attributed to the lower moisture content observed in OutS cheeses, which in turn can be due to the higher ripening temperature occurring in summer. The higher values of friability and graininess observed in OutS cheeses may be also attributed to the effect of the ripening temperature, as a lower water content may have caused a more intense perception of course particles in the mouth (i.e., “grainess”), including the formation of new particles (i.e., “friability”), and a lower perception of cheese adhering to the mouth during mastication (i.e., “adhesivity”).

Both products were well received by consumers as they received scores higher than the central point for overall liking as well as for the liking of appearance, odor/flavor, and texture (Table 6). Consumers were able to distinguish OutS cheeses (i.e., made using milk of grazing animals) from InS cheeses (i.e., made using milk of animals kept indoors). In particular, they expressed higher levels of liking (i.e., overall, appearance, texture, and odor/flavor) for InS cheeses as compared with OutS (*p* < 0.001). In addition, the farm affected the liking of appearance and texture (*p* < 0.05; data not shown). Texture and flavor attributes represent important sets of sensory properties markedly affecting cheese overall quality and consumer liking. Previous studies showed that consumers may express higher levels of liking for products showing characteristics related to freshness [68] with a preference for cheeses with higher perceived moisture and tenderness intensities [62]. Therefore, the higher ripening temperature in summer may have played a negative role in affecting consumer liking for cheeses obtained from grazing animals, and the use of aging facilities with controlled humidity and temperature may be suggested in order to increase the acceptability of pasture-based products.

## 4. Conclusions

The main result of the present study is that the pasture-based, outdoor management system has improved health characteristics of cheese in terms of increased content of PUFA, *cis*-9, *trans*-11 CLA, and *trans*-11 C18:1, along with a reduction of SFA and atherogenic index. Overall, the cheeses obtained from the OutS system showed a higher intensity of almost all sensory attributes, including odor, flavor, taste, and texture descriptors. Concomitantly although all cheeses were scored well above the central point, pasture grazing partly reduced the liking of consumers for the pecorino.

However, changes in the productive process leading to an increment in the water content and softness of the cheeses (i.e., controlled humidity and temperature conditions during ripening) may increase the overall liking of pasture-based products in order to meet the sensory preferences of a higher number of consumers and promote the consumption of healthier foods. The farm of production marginally influenced some of the chemical and sensory characteristics of the cheeses. Overall, the healthier nutritional characteristics, if paired with appropriate sensory characteristics, may strengthen the identity of the mountain cheese while sustaining the income of local people based on upland farming.

## Figures and Tables

**Table 1:** Farm characteristics and ingredients and composition of the diets fed in the barn under the two management systems (raw means ± SD).

Item	InS		OutS	
	Farm A	Farm B	Farm A	Farm B
**Farm Characteristics**				
Usable agricultural area, ha	21	17	21	17
Available grazing areas, ha	-	-	14	8
Lactating ewes, no.	106	78	140	104
**Ingredients of the Diets Fed in the Barn**				
Concentrate ^1^, kg of DM/head per day	0.8	0.8	0.25	0.25
Hay ^2^, kg of DM/head per day	1.8	1.9	0.25	0.40
**Composition of the Diets Fed in Barn**				
Crude protein, % of DM	14.1 ± 0.7	15.4 ± 0.8	14.9 ± 0.5	15.1 ± 0.7
Ether extract, % of DM	2.5 ± 0.4	1.2 ± 0.6	2.6 ± 0.2	1.1 ± 0.4
NDF, % of DM	46.6 ± 1.4	51.7 ± 1.3	40.7 ± 1.1	48.8 ± 1.8
ADF, % of DM	29.6 ± 1.8	23.6 ± 1.5	23.5 ± 0.9	18.2 ± 0.6
NE_L_, MJ/kg of DM	3.6 ± 0.13	3.9 ± 0.17	4.4 ± 0.15	4.5 ± 0.2

^1^ Based on barley, fava beans, oat, and wheat meal. ^2^ Natural meadow and clover hays. InS, indoor system; OutS, outdoor system; DM, dry matter; NDF, neutral detergent fiber; NE_L_, net energy of lactation; SD, standard deviation.

**Table 2:** List of attributes, definitions, and reference frame used by a 12-member panel for Bagnolese pecorino cheese sensory profiling.

Descriptor	Definition	Reference Samples	
		Lower Anchor	Upper Anchor
**Odor**			
Barn	Odor arising from a sheep barn	60 g ricotta cheese	60 g ricotta cheese + 60 g grated pecorino cheese
Hay	Odor arising from hay	200 mL water	5 g hay in 200 mL water
**Flavor**			
Pecorino	Typical flavor of pecorino cheese	cacioricotta cheese	Bagnolese pecorino cheese
Barn	Flavor arising from a sheep barn	60 g ricotta cheese	60 g ricotta cheese + 60 g grated pecorino cheese
**Taste**			
Sweet	Taste elicited by sucrose	8 mL stock solution 100 mL^−1^	20 mL stock solution 100 mL^−1^
Salty	Taste elicited by sodium chloride	1.5 mL stock solution 100 mL^−1^	3 mL stock solution 100 mL^−1^
Acid	Taste elicited by citric acid	8 mL stock solution 10^1^	16 mL stock solution 100 mL^−1^
Bitter	Taste elicited by quinine	4 mL stock solution 100 mL^−1^	8 mL stock solution 100 mL^−1^
Spicy	Taste associated with an irritating or aggressive sensation perceived in the mouth or in the throat	10 g ricotta cheese	10 g ricotta cheese + 0.2 g hot pepper in powder
**Texture**			
Hardness	Highest force required to chew cheese samples	20 g mozzarella cheese	20 g Pecorino cheese ripened 12 months
Friability	Increasing perception of cheese fragments during mastication	20 g Emmental cheese	20 g parmesan cheese ripened 36 months
Grainess	Perception of course particles in the mouth	20 g Fontina cheese	20 g parmesan cheese ripened 36 months
Solubility	Perception of cheese melting in the mouth	20 g Fontina cheese	20 g mini cheese spread
Adhesivity	Effort needed to remove the layer of cheese coating the mouth	20 g mozzarella cheese	20 g Taleggio cheese

**Table 3:** Botanical and chemical compositions of pasture samples (raw means ± SD).

Item	Farm	
	A	B
**Botanical Composition**		
Nonedible biomass,^1^ % DM	12.8 ± 1.2	11.5 ± 1.3
Poaceae, % DM of edible biomass	64.5 ± 3.9	63.8 ± 1.3
*Lolium* spp., % DM of Poaceae	70.8 ± 2.6	65.1 ± 0.6
Other Poaceae,^2^ % DM of Poaceae	29.2 ± 2.6	34.9 ± 0.6
Fabaceae, % DM of edible biomass	22.8 ± 4.2	19.2 ± 4.1
*Trifolium* spp., % DM of Fabaceae	71.1 ± 4.3	61.3 ± 3.8
Other *Fabaceae,*^3^ % DM of Fabaceae	28.9 ± 4.3	38.7 ± 3.8
Other species,^4^ % DM of edible biomass	12.7 ± 1.1	16.9 ± 3.0
**Chemical Composition**		
DM, %	23.8 ± 1.3	22.6 ± 0.6
Crude Protein, % of DM	17.5 ± 0.5	17.2 ± 0.5
Ether extract, % of DM	2.3 ± 0.1	2.5 ± 0.1
NDF, % of DM	47.8 ± 2.0	47.2 ± 1.1
ADF, % of DM	22.3 ± 1.3	25.7 ± 3.0
NE_L_, MJ/kg of DM	5.4 ± 0.5	5.4 ± 0.2

^1^ Nonedible species, mature plants, and dead materials. ^2^ *Bromus* spp., *Arrhenatherum elatius, Avena* spp., *Dactylis glomerata, Hordeum* spp., *Festuca* spp., *Agrostis* spp., *Phleum* spp., *Poa* spp., *Agropyron* spp., and *Trisetum* spp. ^3^ *Medicago* spp., *Lotus corniculatus, Vicia* spp., *Hedysarum coronarium, Onobrychis viciifolia*, and *Anthyllis vulneraria*. ^4^ *Carduus* spp., *Cichorium* spp., *Calendula arvensis, Chrysanthemum coronarium, Mentha* spp., *Achillea* spp., *Borago officinalis, Taraxacum* spp., *Foeniculum vulgare*, *Cirsium* spp., and *Plantago* spp. DM, dry matter; NDF, neutral detergent fiber; ADF, acid detergent fiber; NE_L_, net energy of lactation; SD, standard deviation.

**Table 4:** Milk and cheese chemical composition and color characteristics of cheese (LSM ± SEM) as affected by the management system, farms, and their interaction.

Item	OutS		InS		SEM	Significance ^1^		
	Farm A	Farm B	Farm A	Farm B		Management	Farm	M × F
**Milk**								
Fat, %	6.87	6.92	6.62	7.03	0.10	*	NS	NS
Protein, %	4.80	4.99	4.87	5.21	0.13	+	NS	NS
Lactose, mg/dL	4.80	5.03	4.70	4.73	0.08	NS	*	NS
SCC, log_10_ *n*. cells/mL	5.65	6.29	5.84	6.02	0.19	+	NS	NS
**Cheese**								
DM, %	69.73	72.38	67.43	68.6	0.82	**	*	NS
Ash, % of DM	4.61	4.51	4.31	4.51	0.20	NS	NS	NS
Fat, % of DM	49.55	46.58	46.58	44.35	0.56	***	***	NS
Protein, % of DM	32.18	36.08	33.45	37.30	1.17	NS	**	NS
**Cheese Color**								
L *	72.33	71.88	71.75	70.95	1.26	NS	NS	NS
A *	4.28	5.00	4.60	4.00	0.37	NS	NS	NS
B *	11.10	10.50	11.63	11.08	0.45	NS	NS	NS

^1^ * *p* ≤ 0.05; ** *p* ≤ 0.01 ***; *p* ≤ 0.001; + indicates *p* ≤ 0.10; NS (not significant) indicates *p* > 0.10. OutS, outdoor system; InS, indoor system; M, management; F, farm; DM, dry matter; SCC, somatic cell count; L *, lightness; A *, red-green color; B *, yellow–blue color; LSM, least square mean; SEM, standard error of mean.

**Table 5:** Pecorino cheese fatty acids composition as affected by the management system, the farms, and their interaction (LSM ± SEM).

FA, g/100 g of FA	OutS		InS		SEM	Significance ^1^		
	Farm A	Farm B	Farm A	Farm B		Management	Farm	M × F
C4:0	5.78	4.04	3.97	3.20	0.61	+	NS	NS
C6:0	3.18	2.79	3.25	3.20	0.32	NS	NS	NS
C8:0	2.66	2.52	2.59	2.27	0.10	NS	*	NS
C10:0	6.58	7.41	7.41	7.25	0.16	NS	NS	*
C12:0	3.57	4.22	4.72	4.82	0.19	*	NS	NS
C14:0	11.39	12.09	13.22	13.79	0.12	***	***	NS
C14:1	1.14	1.20	1.19	1.08	0.07	NS	NS	NS
C15:0	1.43	1.43	1.47	1.42	0.06	NS	NS	NS
C16:0	23.58	23.92	27.37	27.60	0.74	***	***	NS
C16:1	1.08	1.29	1.08	1.25	0.05	NS	**	NS
C17:0	0.67	0.78	0.80	0.76	0.06	NS	NS	NS
C17:1	0.42	0.43	0.42	0.39	0.03	NS	NS	NS
C18:0	10.01	10.59	10.66	10.69	0.64	NS	NS	NS
C18:1 n9 *trans*	4.03	3.50	2.96	2.90	0.31	*	NS	NS
C18:1 *trans*-11	0.38	0.35	0.20	0.15	0.02	***	NS	NS
C18: 1 n9 *cis*	17.57	17.36	14.12	15.02	0.47	***	NS	NS
C18:2 n6	1.83	1.72	1.14	0.99	0.08	***	NS	NS
C18:3 n3	1.57	1.31	0.76	0.77	0.17	**	NS	NS
C20:1	0.25	0.23	0.23	0.20	0.02	NS	NS	NS
*Cis*-9 *trans*-11 CLA	2.10	1.93	1.60	1.43	0.19	*	NS	NS
C20:4	0.13	0.12	0.15	0.11	0.01	NS	**	NS
Others	0.66	0.76	0.70	0.71	0.03	NS	*	NS
MUFA	24.87	24.40	20.17	21.00	0.43	***	NS	NS
PUFA	5.64	5.08	3.66	3.31	0.23	*	NS	NS
SFA	68.84	69.79	75.45	74.99	0.42	***	NS	NS
Atherogenic index ^2^	2.38	2.60	3.57	3.60	0.08	***	NS	NS

^1^ * *p* ≤ 0.05; ** *p* ≤ 0.01 ***; *p* ≤ 0.001; + indicates *p* ≤ 0.10; NS (not significant) indicates *p* > 0.10. ^2^ (C12:0 + (4 × C14:0) + C16:0)/unsaturated FA. OutS, outdoor system; InS, indoor system; M, management; F, farm; FA, fatty acids; MUFA, monounsaturated fatty acids; PUFA, polyunsaturated fatty acids; SFA, saturated fatty acids; LSM, least square mean; SEM, standard error of mean.

**Table 6:** Sensory profile, as assessed by a 12-member trained panel, and hedonic scores, as scored by a 100-member untrained consumer panel, of Bagnolese pecorino cheese affected by the management system, the farms, and their interaction (LSM ± SEM).

Descriptor	OutS		InS		SEM	Significance ^1^		
	Farm A	Farm B	Farm A	Farm B		Management	Farm	M × F
**Odour**								
Barn	36.38	72.91	33.36	50.11	3.9	**	***	*
Hay	36.20	49.44	34.84	35.09	4.0	*	*	*
**Flavor**								
Pecorino	43.04	65.07	35.66	51.15	3.7	**	***	NS
Barn	23.18	58.88	24.16	38.89	3.4	**	***	**
**Taste**								
Sweet	7.73	3.27	30.86	14.67	2.5	***	***	*
Salty	35.51	58.36	18.39	39.26	3.0	***	***	NS
Acid	11.96	26.49	16.93	22.41	3.0	NS	**	NS
Bitter	14.29	24.53	13.12	23.22	3.1	NS	**	NS
Spicy	14.38	31.53	10.18	19.65	3.1	*	***	NS
**Texture**								
Hardness	55.33	71.82	21.65	43.85	2.7	***	***	NS
Friability	44.69	55.04	25.02	44.82	3.5	***	***	NS
Grainess	51.18	68.20	23.39	46.33	3.5	***	***	NS
Solubility	23.11	22.04	30.27	25.61	3.5	NS	NS	NS
Adhesivity	26.20	25.67	32.52	33.82	3.4	*	NS	NS
**Hedonic scores**								
Overall liking	6.44	6.13	7.23	7.06	0.21	***	NS	NS
Appearance	6.63	6.18	7.35	7.07	0.19	***	*	NS
Taste/flavor	6.33	6.16	7.03	7.00	0.21	***	NS	NS
Texture	6.53	6.04	7.34	6.89	0.20	***	*	NS

^1^ * *p* ≤ 0.05; ** *p* ≤ 0.01 ***; *p* ≤ 0.001; NS (not significant) indicates *p* > 0.10. OutS, outdoor system; InS, indoor system; M, management; F, farm; LSM, least square mean; SEM, standard error of mean.

## References

[1] Braghieri A., Girolami A., Riviezzi A.M., Piazzolla N., Napolitano F. (2014). Liking of Traditional Cheese and Consumer Willingness to Pay. Ital. J. Anim. Sci..

[2] Uzun P., Serrapica F., Masucci F., Barone C.M.A., Yildiz H., Grasso F., Di Francia A. (2020). Diversity of traditional Caciocavallo cheeses produced in Italy. Int. J. Dairy Technol..

[3] Fox P.F., Cogan T.M., Guinee T.P., McSweeney P.L.H., Fox P.F., Cotter P.D., Everett D.W. (2017). Chapter 25—Factors That Affect the Quality of Cheese. Cheese.

[4] Sabia E., Gauly M., Napolitano F., Cifuni G.F., Claps S. (2020). The effect of different dietary treatments on volatile organic compounds and aromatic characteristics of buffalo Mozzarella cheese. Int. J. Dairy Technol..

[5] Giaccone D., Revello-Chion A., Galassi L., Bianchi P., Battelli G., Coppa M., Tabacco E., Borreani G. (2016). Effect of milk thermisation and farming system on cheese sensory profile and fatty acid composition. Int. Dairy J..

[6] Martin B., Verdier-Metz I., Buchin S., Hurtaud C., Coulon J.-B. (2005). How do the nature of forages and pasture diversity influence the sensory quality of dairy livestock products?. Anim. Sci..

[7] Uzun P., Masucci F., Serrapica F., Napolitano F., Braghieri A., Romano R., Manzo N., Esposito G., Di Francia A. (2018). The inclusion of fresh forage in the lactating buffalo diet affects fatty acid and sensory profile of mozzarella cheese. J. Dairy Sci..

[8] Giorgio D., Di Trana A., Di Napoli M., Sepe L., Cecchini S., Rossi R., Claps S. (2019). Comparison of cheeses from goats fed 7 forages based on a new health index. J. Dairy Sci..

[9] Serrapica F., Uzun P., Masucci F., Napolitano F., Braghieri A., Genovese A., Sacchi R., Romano R., Barone C.M.A., Di Francia A. (2020). Hay or silage? How the forage preservation method changes the volatile compounds and sensory properties of Caciocavallo cheese. J. Dairy Sci..

[10] Alothman M., Hogan S.A., Hennessy D., Dillon P., Kilcawley K.N., O’Donovan M., Tobin J.T., Fenelon M., O’Callaghan T.F. (2019). The “Grass-Fed” Milk Story: Understanding the Impact of Pasture Feeding on the Composition and Quality of Bovine Milk. Foods.

[11] Sabia E., Gauly M., Napolitano F., Serrapica F., Cifuni G.F., Claps S. (2020). Dairy sheep carbon footprint and ReCiPe end-point study. Small Rumin. Res..

[12] Scocco P., Piermarteri K., Malfatti A., Tardella F.M., Catorci A. (2016). Increase of drought stress negatively affects the sustainability of extensive sheep farming in sub-Mediterranean climate. J. Arid. Environ..

[13] Nudda A., Battacone G., Neto O.B., Cannas A., Francesconi A.H.D., Atzori A., Pulina G. (2014). Feeding strategies to design the fatty acid profile of sheep milk and cheese. Rev. Bras. de Zootec..

[14] Cabiddu A., Delgadillo-Puga C., DeCandia M., Molle G., Delgadillo-Puga C. (2019). Extensive Ruminant Production Systems and Milk Quality with Emphasis on Unsaturated Fatty Acids, Volatile Compounds, Antioxidant Protection Degree and Phenol Content. Animals.

[15] Nudda A., McGuire M., Battacone G., Pulina G. (2005). Seasonal Variation in Conjugated Linoleic Acid and Vaccenic Acid in Milk Fat of Sheep and its Transfer to Cheese and Ricotta. J. Dairy Sci..

[16] Todaro M., Bonanno A., Scatassa M.L. (2013). The quality of Valle del Belice sheep’s milk and cheese produced in the hot summer season in Sicily. Dairy Sci. Technol..

[17] Addis M., Fiori M., Riu G., Pes M., Salvatore E., Pirisi A. (2015). Physico-chemical characteristics and acidic profile of PDO Pecorino Romano cheese: Seasonal variation. Small Rumin. Res..

[18] Horwitz W., Association of Official Analytical Chemists (AOAC) (2002). Official Methods of Analysis.

[19] Van Soest P., Robertson J., Lewis B. (1991). Methods for Dietary Fiber, Neutral Detergent Fiber, and Nonstarch Polysaccharides in Relation to Animal Nutrition. J. Dairy Sci..

[20] Sauvant D., Nozière P. (2013). La quantification des principaux phénomènes digestifs chez les ruminants: Les relations utilisées pour rénover les systèmes d’unités d’alimentation énergétique et protéique. INRA Prod. Anim..

[21] Romano R., Masucci F., Giordano A., Spagna Musso S., Naviglio D., Santini A. (2010). Effect of tomato by-products in the diet of Comisana sheep on composition and conjugated linoleic acid content of milk fat. Int. Dairy J..

[22] Ulbricht T., Southgate D. (1991). Coronary heart disease: Seven dietary factors. Lancet.

[23] Masucci F., De Rosa G., Barone C.M.A., Napolitano F., Grasso F., Uzun P., Di Francia A. (2015). Effect of group size and maize silage dietary levels on behaviour, health, carcass and meat quality of Mediterranean buffaloes. Animals.

[24] Murray J., Delahunty C., Baxter I. (2001). Descriptive sensory analysis: Past, present and future. Food Res. Int..

[25] ISO (2012). ISO 8586:2012Sensory Analysis—General Guidelines for the Selection, Training and Monitoring of Selected Assessors and Expert Sensory Assessor.

[26] Albenzio M., Santillo A., Caroprese M., Braghieri A., Sevi A., Napolitano F. (2013). Composition and sensory profiling of probiotic Scamorza ewe milk cheese. J. Dairy Sci..

[27] Di Cagno R., Banks J., Sheehan L., Fox P.F., Brechany E., Corsetti A., Gobbetti M. (2003). Comparison of the microbiological, compositional, biochemical, volatile profile and sensory characteristics of three Italian PDO ewes’ milk cheeses. Int. Dairy J..

[28] Torracca B., Pedonese F., López M.B., Turchi B., Fratini F., Nuvoloni R. (2016). Effect of milk pasteurisation and of ripening in a cave on biogenic amine content and sensory properties of a pecorino cheese. Int. Dairy J..

[29] Braghieri A., Zotta T., Morone G., Piazzolla N., Majlesi M., Napolitano F. (2018). Starter cultures and preservation liquids modulate consumer liking and shelf life of mozzarella cheese. Int. Dairy J..

[30] Markey O., Souroullas K., Fagan C.C., Kliem K.E., Vasilopoulou D., Jackson K.G., Humphries D.J., Grandison A.S., Givens D.I., Lovegrove J.A. (2017). Consumer acceptance of dairy products with a saturated fatty acid–reduced, monounsaturated fatty acid–enriched content. J. Dairy Sci..

[31] Esposito G., Masucci F., Napolitano F., Braghieri A., Romano R., Manzo N., Di Francia A. (2014). Fatty acid and sensory profiles of Caciocavallo cheese as affected by management system. J. Dairy Sci..

[32] Uzun P., Masucci F., Serrapica F., Varricchio M.L., Pacelli C., Claps S., Di Francia A. (2018). Use of mycorrhizal inoculum under low fertilizer application: Effects on forage yield, milk production, and energetic and economic efficiency. J. Agric. Sci..

[33] Sevi A., Albenzio M., Marino R., Santillo A., Muscio A. (2004). Effects of lambing season and stage of lactation on ewe milk quality. Small Rumin. Res..

[34] Sitzia M., Bonanno A., Todaro M., Cannas A., Atzori A.S., Francesconi A.H.D., Trabalza-Marinucci M. (2015). Feeding and management techniques to favour summer sheep milk and cheese production in the Mediterranean environment. Small Rumin. Res..

[35] Todaro M., Dattena M., Acciaioli A., Bonanno A., Bruni G., Caroprese M., Mele M., Sevi A.C., Trabalza-Marinucci M. (2015). Aseasonal sheep and goat milk production in the Mediterranean area: Physiological and technical insights. Small Rumin. Res..

[36] Bianchi L., Casoli C., Pauselli M., Budelli E., Caroli A., Bolla A., Duranti E. (2004). Effect of somatic cell count and lactation stage on sheep milk quality. Ital. J. Anim. Sci..

[37] Fthenakis G. (1994). Prevalence and aetiology of subclinical mastitis in ewes of Southern Greece. Small Rumin. Res..

[38] Fthenakis G. (1996). Somatic cell counts in milk of Welsh-Mountain, Dorset-Horn and Chios ewes throughout lactation. Small Rumin. Res..

[39] Carpino S., Mallia S., La Terra S., Melilli C., Licitra G., Acree T., Barbano D.M., Van Soest P. (2004). Composition and Aroma Compounds of Ragusano Cheese: Native Pasture and Total Mixed Rations. J. Dairy Sci..

[40] Segato S., Balzan S., Elia C.A., Lignitto L., Granata A., Magro L., Contiero B., Andrighetto I., Novelli E. (2007). Effect of period of milk production and ripening on quality traits of Asiago cheese. Ital. J. Anim. Sci..

[41] Cozzi G., Ferlito J., Pasini G., Contiero B., Gottardo F. (2009). Application of Near-Infrared Spectroscopy as an Alternative to Chemical and Color Analysis to Discriminate the Production Chains of Asiago d’Allevo Cheese. J. Agric. Food Chem..

[42] Cardinault N., Doreau M., Poncet C., Nozière P. (2006). Digestion and absorption of carotenoids in sheep given fresh red clover. Anim. Sci..

[43] Palmquist D.L. (2007). Milk Fat: Origin of Fatty Acids and Influence of Nutritional Factors Thereon. Advanced Dairy Chemistry Volume 2 Lipids.

[44] Bauman D.E., Griinari J.M. (2003). Nutritional regulation of milk fat synthesis. Annu. Rev. Nutr..

[45] MacGibbon A., Taylor M.W. (2007). Composition and Structure of Bovine Milk Lipids. Advanced Dairy Chemistry Volume 2 Lipids.

[46] Mele M. (2009). Designing milk fat to improve healthfulness and functional properties of dairy products: From feeding strategies to a genetic approach. Ital. J. Anim. Sci..

[47] Dewhurst R.J., Shingfield K., Lee M., Scollan N. (2006). Increasing the concentrations of beneficial polyunsaturated fatty acids in milk produced by dairy cows in high-forage systems. Anim. Feed. Sci. Technol..

[48] Trani A., Gambacorta G., Loizzo P., Cassone A., Faccia M. (2016). Short communication: Chemical and sensory characteristics of Canestrato di Moliterno cheese manufactured in spring. J. Dairy Sci..

[49] Altomonte I., Conte G., Serra A., Mele M., Cannizzo L., Salari F., Martini M. (2019). Nutritional characteristics and volatile components of sheep milk products during two grazing seasons. Small Rumin. Res..

[50] Caprioli G., Nzekoue F.K., Fiorini D., Scocco P., Trabalza-Marinucci M., Acuti G., Tardella F.M., Sagratini G., Catorci A. (2019). The effects of feeding supplementation on the nutritional quality of milk and cheese from sheep grazing on dry pasture. Int. J. Food Sci. Nutr..

[51] Fusaro I., Giammarco M., Odintsov Vaintrub M., Chincarini M., Manetta A.C., Mammi L.M.E., Palmonari A., Formigoni A., Vignola G. (2020). Effects of three different diets on the fatty acid profile and sensory properties of fresh Pecorino cheese “Primo Sale”. Asian-Australasian J. Anim. Sci..

[52] Gómez-Cortés P., Frutos P., Mantecon A.R., Juárez M., De La Fuente M.A., Hervás G. (2008). Addition of Olive Oil to Dairy Ewe Diets: Effect on Milk Fatty Acid Profile and Animal Performance. J. Dairy Sci..

[53] Gómez-Cortés P., Frutos P., Mantecon A.R., Juárez M., De La Fuente M., Hervás G. (2008). Milk Production, Conjugated Linoleic Acid Content, and In Vitro Ruminal Fermentation in Response to High Levels of Soybean Oil in Dairy Ewe Diet. J. Dairy Sci..

[54] Gómez-Cortés P., Bach A., Luna P., Juárez M., De La Fuente M.A. (2009). Effects of extruded linseed supplementation on n-3 fatty acids and conjugated linoleic acid in milk and cheese from ewes. J. Dairy Sci..

[55] Gómez-Cortés P., De La Fuente M., Toral P.G., Frutos P., Juárez M., Hervás G. (2011). Effects of different forage:concentrate ratios in dairy ewe diets supplemented with sunflower oil on animal performance and milk fatty acid profile. J. Dairy Sci..

[56] Toral P.G., Frutos P., Hervás G., Gómez-Cortés P., Juarez M., De La Fuente M. (2010). Changes in milk fatty acid profile and animal performance in response to fish oil supplementation, alone or in combination with sunflower oil, in dairy ewes. J. Dairy Sci..

[57] Toral P.G., Gómez-Cortés P., Frutos P., de la Fuente M.A., Juárez M., Hervás G., Ranilla M.J., Carro M.D., Ben Salem H., Morand-Fehr P. (2011). Milk production and fatty acid profile after three weeks of diet supplementation with sunflower oil and marine algae in dairy ewes. Challenging Strategies to Promote the Sheep and Goat Sector in the Current Global Context.

[58] Vecchio R., Lombardi A., Cembalo L., Caracciolo F., Cicia G., Masucci F., Di Francia A. (2016). Consumers’ willingness to pay and drivers of motivation to consume omega-3 enriched mozzarella cheese. Br. Food J..

[59] Collomb M., Bütikofer U., Sieber R., Jeangros B., Bosset J.-O. (2002). Correlation between fatty acids in cows’ milk fat produced in the Lowlands, Mountains and Highlands of Switzerland and botanical composition of the fodder. Int. Dairy J..

[60] Falchero L., Lombardi G., Gorlier A., Lonati M., Odoardi M., Cavallero A. (2010). Variation in fatty acid composition of milk and cheese from cows grazed on two alpine pastures. Dairy Sci. Technol..

[61] Kelsey J., Corl B., Collier R., Bauman D. (2003). The Effect of Breed, Parity, and Stage of Lactation on Conjugated Linoleic Acid (CLA) in Milk Fat from Dairy Cows. J. Dairy Sci..

[62] Braghieri A., Piazzolla N., Romaniello A., Paladino F., Ricciardi A., Napolitano F. (2015). Effect of adjuncts on sensory properties and consumer liking of Scamorza cheese. J. Dairy Sci..

[63] Khattab A.R., Guirguis H.A., Tawfik S.M., Farag M.A. (2019). Cheese ripening: A review on modern technologies towards flavor enhancement, process acceleration and improved quality assessment. Trends Food Sci. Technol..

[64] Farruggia A., Pomiès D., Coppa M., Ferlay A., Verdier-Metz I., Le Morvan A., Bethier A., Pompanon F., Troquier O., Martin B. (2014). Animal performances, pasture biodiversity and dairy product quality: How it works in contrasted mountain grazing systems. Agric. Ecosyst. Environ..

[65] Coppa M., Verdier-Metz I., Ferlay A., Pradel P., Didienne R., Farruggia A., Montel M.-C., Martin B. (2011). Effect of different grazing systems on upland pastures compared with hay diet on cheese sensory properties evaluated at different ripening times. Int. Dairy J..

[66] Sacchi R., Marrazzo A., Masucci F., Di Francia A., Serrapica F., Genovese A. (2020). Effects of Inclusion of Fresh Forage in the Diet for Lactating Buffaloes on Volatile Organic Compounds of Milk and Mozzarella Cheese. Molecules.

[67] McSweeney P.L.H. (2004). Biochemistry of cheese ripening. Int. J. Dairy Technol..

[68] Lappalainen R., Kearney J., Gibney M. (1998). A pan EU survey of consumer attitudes to food, nutrition and health: An overview. Food Qual. Preference.

